# Social exclusion increases interpersonal distance through need threat: buffering and amplifying roles of social identification and rejection sensitivity

**DOI:** 10.3389/fpsyg.2026.1881465

**Published:** 2026-09-08

**Authors:** Juanjuan Wei, Zehao Qian

**Affiliations:** 1 China Key Laboratory of Aviation Psychology and Human-Machine Ergonomics, Civil Aviation Flight University of China, Guanghan, Sichuan, China; 2 Sichuan Provincial Engineering Research Center of Domestic Civil Aircraft Flight and Operation Support, Civil Aviation Flight University of China, Guanghan, Sichuan, China

**Keywords:** interpersonal distance, need threat, rejection sensitivity, social exclusion, social identification

## Abstract

**Introduction:**

Although social exclusion influences interpersonal distance preferences, the mechanisms underlying this association remain unclear. The present study addressed this gap by examining whether exclusion-induced fundamental need threat accounts for the association between social exclusion and interpersonal distance, and whether social identification and rejection sensitivity shape this process among Chinese college students.

**Methods:**

A total of 396 participants reported social identification and rejection sensitivity, and completed measures of interpersonal distance at baseline and following inclusion and exclusion conditions manipulated via the Cyberball paradigm, with perceived need threat assessed after each condition.

**Results:**

Results showed that social exclusion increased perceived need threat and preferred interpersonal distance relative to both the baseline and the inclusion conditions. Need threat mediated the association between social exclusion and interpersonal distance, with stronger effects observed among individuals low in social identification and high in rejection sensitivity.

**Discussion:**

These findings not only provide evidence that social exclusion shapes interpersonal distance through exclusion-induced need threat but also offer a new lens for understanding how interpersonal boundaries are regulated in response to social threat, highlighting the role of individual differences in this process.

## 1 Introduction

Interpersonal distance (IPD) is conceptualized as the physical space individuals maintain between themselves and others during social interaction, constituting a fundamental aspect of proxemic behavior (Hayduk, 1978, 1983). The regulation of IPD reflects a dynamic process through which individuals continuously adjust interpersonal boundaries in response to contextual and social cues. The observable variations in distance thus reflect underlying relational intentions. Specifically, closer distances are generally associated with approach-oriented and affiliative motivations, reflecting perceived acceptance and interpersonal openness, whereas greater distances are more commonly linked to avoidance and social disengagement (Argyle and Dean, 1965; Hall and Hall, 1966; Hayduk, 1983). In this vein, maintaining an optimal level of IPD is essential for effective social functioning, while deviations from this optimal range can impair interpersonal interactions: excessive proximity often elicits discomfort and heightened physiological arousal, whereas excessive distance tends to impede communication efficiency and relational engagement (Kroczek et al., 2020; Candini et al., 2021). IPD regulation thus entails balancing competing interpersonal motives: the need to approach others to facilitate social connection and communication, and the desire to maintain personal safety and bodily integrity through adequate spatial boundaries (Hayduk, 1983; Siegman and Feldstein, 2014). This balance is highly sensitive to situational cues: perceived threat or discomfort typically leads to increased IPD, whereas low-threat or comfortable contexts facilitate closer proximity (Candini et al., 2017, 2019; Hayduk, 1978; Gessaroli et al., 2013; Massaccesi et al., 2021). For example, individuals maintain greater distances from avatars displaying angry facial expressions compared to those exhibiting neutral or positive expressions (Ruggiero et al., 2017).

In addition to situational influences, IPD preferences are also shaped by demographic and cultural factors, including age, gender, and cultural background (Hayduk, 1983; Iachini et al., 2016; Pedersen and Heaston, 1972; Pollatos et al., 2015; Sorokowska et al., 2017). Age-related changes suggest that the capacity to regulate appropriate IPD develops early in life, with preferred distances generally increasing with age (Aiello and De Carlo Aiello, 1974; Pagan and Aiello, 1982). Gender differences indicate that individuals tend to maintain shorter distances from female targets than from male targets (Iachini et al., 2016), and cross-cultural research documents consistent variation in proxemic norms across societies (Sorokowska et al., 2017; Wei et al., 2024b). Although these factors contribute to baseline variations in IPD preferences, the regulation of IPD is also highly influenced by relational experiences that shape expectations about social interaction. Among these, social exclusion may be particularly influential, as it threatens perceived belongingness and relational bonds, thereby shaping how interpersonal boundaries are regulated in subsequent encounters.

Social exclusion refers to experiences in which individuals are ignored, rejected, or marginalized by others, resulting in perceived separation from a social group (Büttner et al., 2025; Williams, 2007; Wesselmann et al., 2016). Extensive research indicates that such exclusionary experiences are associated with a range of negative psychological outcomes, including impairments in mental health, meaning in life, and well-being (Akyol Güner and Das Gecim, 2023; Dahlberg and McKee, 2018; Filia et al., 2025; Sommer and Bernieri, 2015; Stillman et al., 2009). Beyond these outcomes, social exclusion also influences how individuals regulate their IPD in subsequent social interactions. Consistent with the multimotive model of rejection (Smart Richman and Leary, 2009), social exclusion can activate both affiliative and self-protective motives, leading individuals to either reduce IPD to re-establish social connection (Knowles et al., 2014; Pollatos et al., 2015) or increase it as a form of interpersonal withdrawal (Chen et al., 2023). These divergent responses suggest that the effect of social exclusion on IPD preferences is context-dependent and may reflect variations in the extent to which exclusion threatens fundamental psychological needs. Accordingly, understanding the psychological processes underlying such variation is critical for elucidating how social exclusion shapes the regulation of IPD.

A central proximal process may involve exclusion-induced threat to fundamental psychological needs. The temporal need-threat framework proposes that exclusion undermines core psychological needs, including belongingness, self-esteem, perceived control, and meaningful existence, which are essential for maintaining adaptive social functioning (Williams, 2007; Zadro et al., 2004). When these needs are disrupted, individuals tend to experience a psychologically aversive state characterized by feelings of relational insecurity, loss of perceived agency, and threats to self-related resources. Consistent with this view, empirical research demonstrates that need threat induced by exclusion, rather than exclusion per se, elicits pronounced affective and cognitive reactions, including increased anxiety (Leary, 1990), depression (Kavakli, 2021), social pain (MacDonald and Leary, 2005), anger (Chow et al., 2008), and loneliness (Dahlberg et al., 2022). These emotional reactions are often accompanied by shifts in social cognition, such as declines in self-esteem (Leary et al., 1995), belongingness (Li and Jiang, 2018; Tetzlaff and Rothe-Wulf, 2025), and interpersonal trust (Pellegrini et al., 2021). Indeed, this state is not limited to affective or social-cognitive responses but functions as a motivational signal, indicating a discrepancy between current interpersonal conditions and fundamental psychological needs. Within this perspective, individuals may engage in various forms of regulatory behavior aimed at restoring psychological equilibrium (Baumeister et al., 2002; Baumeister et al., 2005; Eisenberger et al., 2003; Perchtold-Stefan et al., 2024).

The multimotive model of rejection (Smart Richman and Leary, 2009) further suggests that individuals may respond to exclusion-induced need threat in different ways depending on situational conditions. When opportunities for relational repair are available, individuals may engage in affiliative behaviors (Williams, 2009), such as helping others (Baumeister et al., 2007; Williams and Sommer, 1997), conforming to group norms (Williams et al., 2000), or seeking alternative social connections (Maner et al., 2007). In contrast, when exclusion persists and fundamental needs remain unmet, individuals may exhibit more defensive responses, including social withdrawal, disengagement, and aggression (Brinker et al., 2023; Chen et al., 2023; Perchtold-Stefan et al., 2024; Twenge et al., 2001, 2007; Twenge and Campbell, 2003; Warburton et al., 2006). Such defensive regulation may extend beyond overt social behaviors to the regulation of interpersonal boundaries, including how individuals manage their distance from others. Prior evidence has shown that IPD preferences vary as a function of perceived threat (Lloyd, 2009; Wei et al., 2024a). Within this context, exclusion-induced need threat represents a theoretically relevant form of psychological threat that may be associated with individuals’ regulation of interpersonal proximity. However, whether this specific form of threat is linked to IPD regulation remains unclear. Accordingly, examining whether exclusion-induced need threat contributes to variation in IPD may provide important insight into how psychological responses to exclusion are reflected in the regulation of interpersonal space.

Although social exclusion influences IPD preferences, its effects are not uniform across individuals (Chen et al., 2023; Knowles et al., 2014; Pollatos et al., 2015). Such variability may be related to differences in socio-affective dispositions, particularly those reflecting interpersonal resilience or vulnerability. Among these, social identification and rejection sensitivity have been identified as relevant individual difference factors associated with behavioral and emotional responses to social threat (Traversa et al., 2026). Understanding how these individual differences shape responses to exclusion is critical for explaining variability in IPD regulation. Although exclusion-induced need threat represents a common psychological consequence of social exclusion, social identification and rejection sensitivity may influence how such threat is interpreted, regulated, and translated into interpersonal behavior. Consequently, individuals may differ substantially in the extent to which threatened needs are translated into defensive interpersonal responses. From this perspective, social identification and rejection sensitivity may help explain why similar exclusion experiences result in different patterns of IPD regulation. More broadly, examining these factors may contribute to a more integrated understanding of previously inconsistent findings regarding the effects of social exclusion on IPD.

Social identification (SI) reflects the extent to which individuals incorporate group membership into their self-concept and derive affective attachment and psychological security from that membership (Miller et al., 2015; Thomas et al., 2017; Turner et al., 1987). Social identity theory (Tajfel and Turner, 2010; Turner et al., 1987) posits that group identification constitutes a core component of the self, enabling individuals to draw upon group-based psychological resources to cope with social threats. These resources contribute to maintaining belongingness and self-integrity, thereby shaping the appraisal of exclusion-induced need threat. Consequently, individuals with higher SI may perceive exclusion-induced need threat as less psychologically threatening, thereby reducing the activation of avoidance-oriented regulatory responses. Empirical evidence further indicates that higher SI is associated with shorter IPD toward others, greater persistence in social engagement following exclusion, and lower psychological distress, partly through reduced perceptions of exclusion (Novelli et al., 2010; Traversa et al., 2026; Wei et al., 2024a). In line with the rejection-identification model, ingroup identification can facilitate psychological adjustment in the context of rejection by providing compensatory belongingness and social support (Branscombe et al., 1999). These findings suggest that SI may attenuate defensive responses to exclusion-induced need threat.

University students typically define themselves through multiple social group memberships, including family and peer-based networks. From a social identity perspective, these group memberships vary in their integration into the self-concept and their situational accessibility (Tajfel and Turner, 2010; Turner et al., 1987), which may influence the psychological resources they provide under conditions of social threat. Family identification is generally grounded in stable and enduring relational bonds that offer relatively consistent emotional support across contexts. In contrast, roommate identification is embedded in shared living environments and frequent daily interactions, making it a more proximal and immediately accessible source of belonging during university years (Erb et al., 2014; Koerner and Fitzpatrick, 2004). These distinctions suggest that identification with family and roommates may differentially shape individuals’ responses to exclusion-induced need threat.

Rejection sensitivity (RS) is a cognitive-affective disposition characterized by anxious expectations of rejection and a heightened tendency to perceive and overreact to rejection cues in interpersonal contexts (Downey and Feldman, 1996; Downey et al., 1997, 2004; Feldman and Downey, 1994; Pietrzak et al., 2005). Developmental accounts suggest that RS emerges from repeated experiences of unmet belongingness needs, which foster chronically accessible expectations of rejection across social situations (Levy et al., 2001). Once established, these expectations bias attentional and interpretive processes, increasing the likelihood that ambiguous or even neutral social cues are construed as rejection signals (Bosk et al., 2026; Downey and Feldman, 1996). Such biased processing elicits heightened negative affect (e.g., anxiety and anger) and self-referential cognitions (e.g., self-blame), which in turn promote maladaptive interpersonal responses, including hostility and social withdrawal (Downey et al., 2004; Gao et al., 2017; Watson and Nesdale, 2012; Zimmer-Gembeck and Nesdale, 2013). Over time, these responses reinforce rejection-related expectations, forming a self-perpetuating cycle of heightened sensitivity to interpersonal threat (Meehan et al., 2019; Pietrzak et al., 2005).

Within the context of social exclusion, RS is likely to shape how individuals respond to exclusion-induced need threat. Empirical evidence indicates that individuals higher in RS exhibit more avoidance-oriented responses following exclusion, including disengagement from interaction partners and reduced willingness for interpersonal contact (Ayduk et al., 2001; Meehan et al., 2018; Schaan et al., 2020; Zimmer-Gembeck, 2015). These responses indicate that individuals higher in RS tend to regulate threatened needs in a defensive manner aimed at minimizing further exposure to potential rejection (Lang et al., 1990, 2002). Given that IPD constitutes a behavioral indicator of approach-avoidance tendencies, heightened RS may strengthen defensive interpersonal regulation following exclusion-induced need threat through amplified threat appraisals and biased interpretations of interpersonal risk cues, resulting in preferences for greater IPD. Accordingly, RS is expected to moderate the association between exclusion-induced need threat and IPD preferences, such that this association is stronger among individuals higher in RS.

Despite substantial evidence linking social exclusion to IPD preferences (Knowles et al., 2014; Pollatos et al., 2015; Smart Richman and Leary, 2009), prior research has primarily considered exclusion-induced need threat and IPD as distinct outcomes, leaving the psychological processes through which need threat shapes interpersonal behavior insufficiently specified. The present study addresses this gap by proposing that exclusion-induced need threat functions as a proximal psychological mechanism underlying variation in IPD following exclusion. In this view, IPD may reflect behavioral regulation in response to threatened fundamental needs, providing further evidence that such needs are embodied in spatial patterns of interpersonal behavior.

Thus, the first aim of the study is to replicate and extend prior findings on the effects of social exclusion on IPD preferences among Chinese college students, while considering target gender as a contextual factor (Knowles et al., 2014; Pollatos et al., 2015). The second aim is to examine whether exclusion-induced need threat accounts for the association between social exclusion and IPD preferences. The third aim is to investigate whether individual differences in social identification and rejection sensitivity moderate the association between need threat and IPD preferences, reflecting protective and vulnerability processes in interpersonal safety management (Perry et al., 2013; Schaan et al., 2020; Wei et al., 2024a; Zimmer-Gembeck, 2015).

To achieve these aims, participants first completed measures of social identification and rejection sensitivity, followed by a baseline assessment of IPD using a computerized version of the Interpersonal Visual Analogue Scale (i.e., IVAS; Iachini et al., 2016, 2021; Wei et al., 2024a). Social inclusion and exclusion were then experimentally manipulated using the Cyberball paradigm, after which participants reported perceived need threat via the Need Threat Scale and completed the IVAS following each condition. Given that most prior studies using the IVAS task have been conducted with European adults, a pilot study was performed to ensure the cultural appropriateness of the target stimuli for Chinese participants.

## 2 Pilot study

We selected four photographs depicting targets that varied in gender (two females and two males). To pre-test the recognition of targets, a sample of Chinese participants was asked to assess the extent to which the targets are perceived in terms of gender, age, and ethnicity.

## 3 Method

### 3.1 Participants

*A priori* power analysis was conducted using G*Power (Faul et al., 2009) for a one-way repeated-measures ANOVA with four within-subject levels, revealing that 36 participants would yield 0.95 statistical power to detect a medium-sized effect of 0.25. To ensure adequate statistical precision and account for potential data exclusions, a total of 85 participants (56 male, *M* ± *SD*_age_ = 19.55 ± 0.84 years, age range = 18–22 years) were recruited from a university in China. Participants had normal or corrected-to-normal vision. The study was approved by the Ethics Committee of China Key Laboratory of Aviation Psychology and Human-Machine Ergonomics, and informed consent was obtained prior to participation.

### 3.2 Materials

Four photographs portraying young adult Asian individuals were sourced from the publicly accessible online image repository (https://image.baidu.com/), consisting of 2 females and 2 males. All images were scaled to a resolution of 1,280 × 720 pixels.

### 3.3 Procedure

The study was conducted online using the Qualtrics platform. Participants were presented with 4 pictures, displayed one at a time in a randomized order at the center of a computer screen. Each picture depicted a target varying in three attributes: gender (female or male), age category (adolescent, young adult: 18–25 years, or adult: older than 26 years), and ethnic background (European, Asian, African, or Other). For each picture, participants rated the extent to which the target belonged to each category within these three dimensions (i.e., gender, age, and ethnicity), yielding a total of nine items per picture. All ratings were provided on a 5-point Likert-type scale ranging from 1 (“not at all”) to 5 (“completely”).

## 4 Results

Paired-samples t-tests and two sets of one-way repeated-measures ANOVAs were conducted to examine whether the four pictures were clearly identifiable in terms of their gender, age, and ethnicity.

For gender, the target category was defined as the category corresponding to the attribute depicted in each photograph (female vs. male). Paired-samples t-tests compared ratings for the target category versus the non-target category. Ratings for the target category were significantly higher than those for the non-target category across all four pictures. For Pictures 1 and 2 (female target category), ratings for female were higher than those for male, *t*(84) = 52.93, *p* < 0.001, and *t*(84) = 46.00, *p* < 0.001, respectively, whereas for Pictures 3 and 4 (male target category), ratings for male were significantly higher than those for female, *t*(84) = 75.42, *p* < 0.001, and *t*(84) = 55.39, *p* < 0.001, respectively. Moreover, by using a t-test against 3 (i.e., the value which corresponds to the midpoint of the scale), we found that ratings consistently deviated from the midpoint across all pictures (all *ps* <0.001). For pictures 1 and 2, ratings for females were significantly above the midpoint, whereas ratings for males were significantly below it (all *ps* <0.001). In contrast, for Pictures 3 and 4, ratings for males were significantly above the midpoint, whereas ratings for females were significantly below it (all *ps* <0.001). This evidence suggests that each picture was reliably identified in terms of gender.

For age, separate one-way repeated-measures ANOVAs were conducted for each picture across the three age-category ratings (adolescent, young adult, adult), revealing a significant main effect of age category for all four pictures, *Fs*(2, 168) = 37.07–97.16, all *ps* < 0.001 (η_p_^2^ = 0.31–0.54). Bonferroni-corrected comparisons indicated that, for each picture, ratings for young adults were significantly higher than those for adolescents and adults (all *ps* < 0.001). One-sample t-tests were conducted against the scale midpoint (3), indicating that ratings significantly differed from the midpoint across all age categories (all *ps* < 0.001). For each picture, ratings for young adults were significantly above the midpoint, whereas ratings for adolescents and adults were significantly below it (all *ps* < 0.001). These findings indicate that each picture was reliably categorized as depicting a young adult individual.

For ethnicity, separate one-way repeated-measures ANOVAs were conducted for each picture across the four ethnicity-category ratings (European, Asian, African, Other), revealing a significant main effect of ethnicity category for all four pictures, *Fs*(3, 252) = 1214.88–2004.95, all *ps* < 0.001 (η_p_^2^ = 0.97–0.98). Bonferroni-corrected comparisons consistently showed that, for each picture, ratings for Asian were significantly higher than those for European, African and Other (all *ps* < 0.001). One-sample t-tests were performed against the scale midpoint (3), indicating that ratings significantly deviated from the midpoint across all ethnicity categories (all *ps* <0.001). For each picture, ratings for Asian were significantly above the midpoint, whereas ratings for European, African, and Other were significantly below it (all *ps* <0.001). These results suggest that each picture was reliably identifiable as an Asian individual.

Taken together, these findings indicate that the targets depicted in the pictures were notably identifiable as either female or male, as young adults, and as Asian individuals.

## 5 Main Study

### 5.1 Method

#### 5.1.1 Participants and design

A total of 400 undergraduate students were initially recruited from a public university in the Sichuan region of China between January and November 2025. Using a random sampling procedure, 11 classes were selected for participation. Participants were required to meet the following inclusion criteria: (a) being between 18 and 24 years of age and (b) reporting no medical conditions that could interfere with task performance. Four participants failed to complete the survey and were therefore excluded from the analyses, resulting in a final sample of 396 participants (69 females, age range = 18–24 years; *M* ± *SD*_age_ = 19.31 ± 1.23 years).

The study protocol was approved by the Ethics Committee of China Key Laboratory of Aviation Psychology and Human-Machine Ergonomics. All recruitment and data collection procedures were conducted in accordance with the ethical standards of the Declaration of Helsinki (World Medical Association, 2013). Informed consent was digitally obtained from participants. In addition, participants were further informed about the anonymous and voluntary nature of the survey and the possibility of withdrawing from the study at any time.

#### 5.1.2 Materials and procedure

##### 5.1.2.1 Social identification with roommates and family

Participants’ identification with roommates and family was measured by the Group Identification Scale (Thomas et al., 2017; Wei et al., 2024a). Each subscale comprised 6 items with a response Likert-type ranging from 1 (strongly disagree) to 5 (strongly agree). These items capture cognitive, emotional, and behavioral dimensions of participants’ SI: i.e., “Belonging to the group of my roommates/family is very important for who I am”. High scores of SI indicate that such groups are important to individuals’ self-definition (Tajfel and Turner, 2010). Cronbach’s Alphas were assessed for each context (SI *α* = 0.86, SI with roommates *α* = 0.86, SI with family *α* = 0.88).

##### 5.1.2.2 Rejection sensitivity

The Rejection Sensitivity Questionnaire (RSQ) was administered to assess individuals’ tendency to anxiously expect and readily perceive interpersonal rejection (Downey and Feldman, 1996). Participants completed the Chinese version of the RSQ, which consists of 16 interpersonal scenarios (e.g., “You are asking a teacher at your university for help in a difficult situation”). For each scenario, participants provided two ratings: their level of concern about receiving help and their optimism about not being rejected, both assessed on 6-point Likert scales (1 = not concerned at all/highly unlikely, 6 = highly concerned/very likely), with higher scores indicating greater concern and greater perceived likelihood of acceptance, respectively (Zhao et al., 2012). Following standard RSQ scoring procedures, optimism ratings were reverse-scored, and a composite RS score for each scenario was computed by multiplying the concern rating by the reverse-scored optimism rating. An overall RS score was obtained by averaging these composite scores across the 16 scenarios, with higher scores reflecting greater RS (Ayduk et al., 2001). The scale demonstrated good internal consistency in the present study (Cronbach’s α = 0.90).

##### 5.1.2.3 Interpersonal distance

Interpersonal distance was measured using the Interpersonal Visual Analogue Scale (IVAS; Iachini et al., 2016, 2021; Wei et al., 2024b), administered online via the Qualtrics survey platform. The four photographs validated in the pilot study were used as stimulus materials: one female and one male photograph labeled “YOU/你”, representing the participant, and one female and one male photograph labeled “OTHER TARGET/其他人”, representing the target individual. The participant photograph was matched to the participants’ own gender, whereas both female and male target photographs were shown to all participants. The same photograph was never used as both the participant and target stimulus.

**Figure 1:**
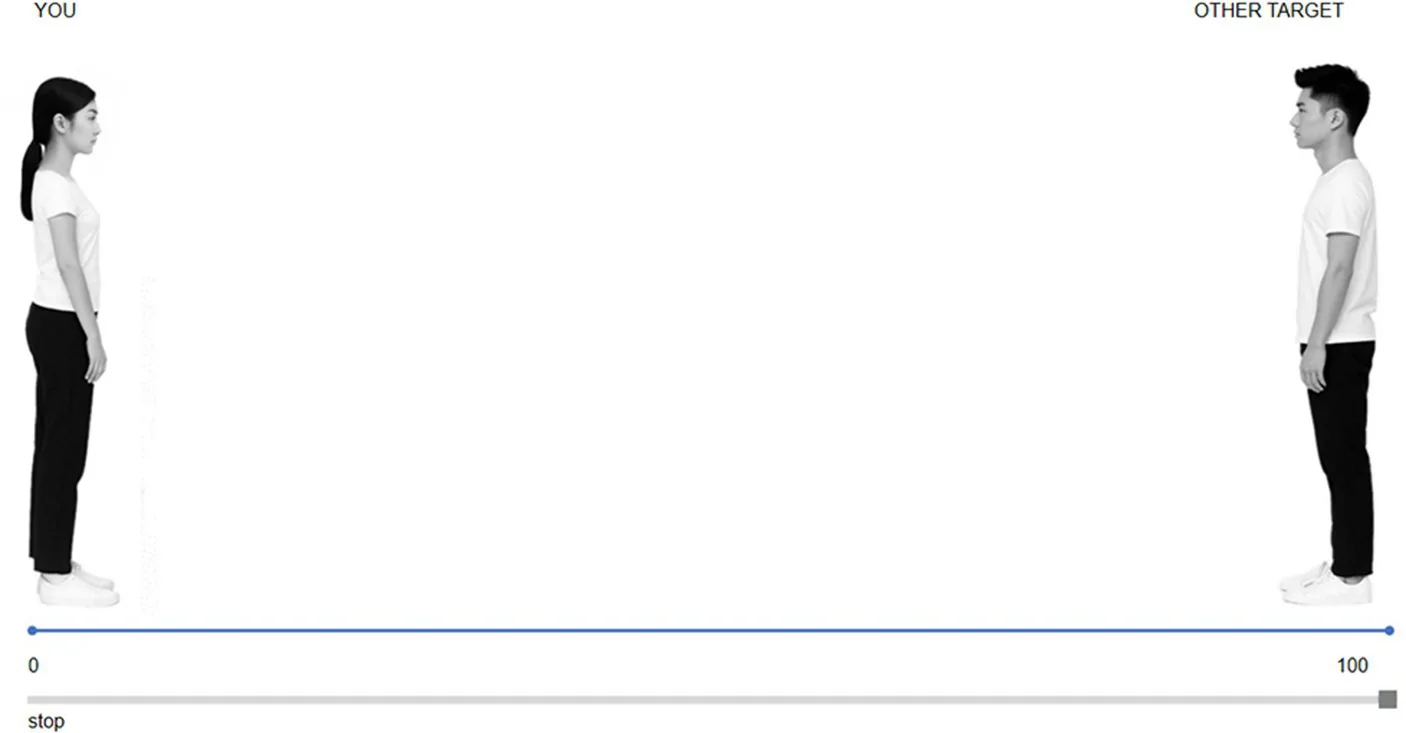
The participant (“YOU/你”) and the target (“OTHER TARGET/其他人”) are positioned at opposite side of a horizontal line. Participants indicated their preferred interpersonal distance by moving a rectangular slider along the grey line, ranging from 100 to 0, with greater values indicating a greater preferred interpersonal distance from the target.

In each trial, participants were presented with an image depicting the participant and the target positioned at opposite ends of a horizontal line, with an initial distance fixed at 100 mm (see Figure 1). Participants were instructed to imagine themselves as the person labeled “YOU/你”, standing still, while the target individual approached them from the front. Then, they were asked to indicate their preferred IPD by moving a rectangular slider along the line to determine the point at which the approaching target should stop. Each participant completed four trials in total, with two trials per target gender, and the order of target presentation was randomized across participants.

##### 5.1.2.4 Exclusion

###### 5.1.2.4.1 Cyberball task

**Figure 2:**
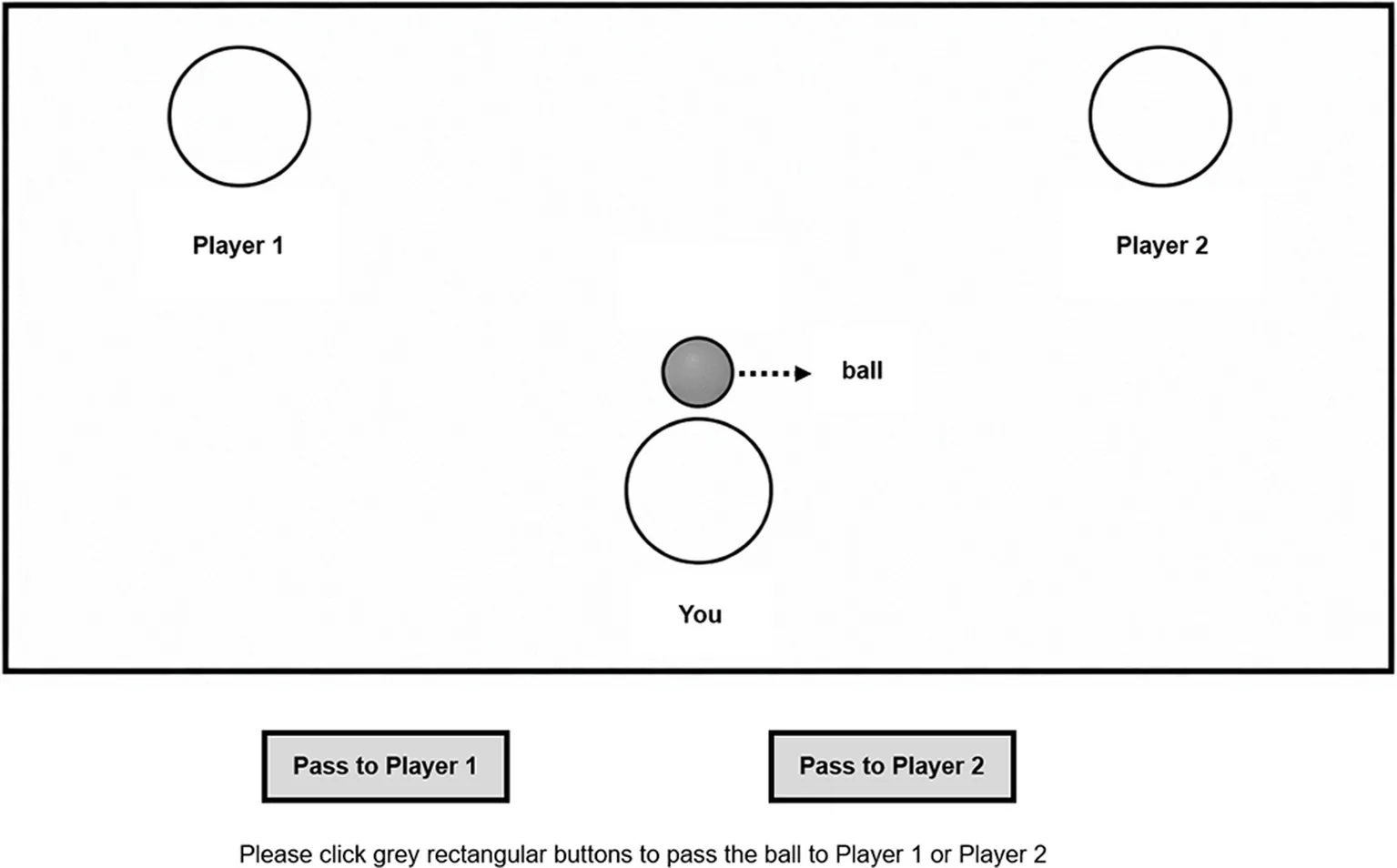
Cyberball task. Participants pass the ball to one of two players by clicking the corresponding on-screen button when receiving the ball.

Cyberball, a computerized virtual ball-tossing game, was used to experimentally operationalize social inclusion and exclusion (Williams et al., 2000; Williams and Jarvis, 2006). The task consisted of 30 ball tosses among three players: the participant and two computerized players (Figure 2). When participants received the ball, they were instructed to pass it to Player 1 or Player 2 by clicking the corresponding grey rectangular button on the screen. All participants completed both the inclusion and exclusion conditions in a fixed sequence, with the inclusion condition administered first, followed by the exclusion condition. In the inclusion condition, ball tosses were evenly distributed among the three players, with participants receiving the ball on one third of the tosses (10 out of 30). In the exclusion condition, participants received the ball twice at the beginning of the game (once from each computerized player) and were subsequently excluded from further play, with the remaining tosses exchanged only between the two computerized players. To minimize demand characteristics, participants were informed that the task was designed to assess mental visualization abilities. Cyberball 4.0 was implemented via Qualtrics by using the script obtained from www.cyberball.wikispaces.com.

###### 5.1.2.4.2 Need threat scale (NTS)

Following the Cyberball task, participants in both the inclusion and exclusion conditions completed the 12-item Need-Threat Scale to assess threats to four fundamental needs: belonging (e.g., “I felt like an outsider during the game”), self-esteem (e.g., “I felt good about myself during the game”), meaningful existence (e.g., “I felt nonexistent during the game”), and control (e.g., “I felt I had the ability to influence the course of the game”; Jamieson et al., 2010; Williams et al., 2000, 2002). Each need was measured using three items. Participants rated each item on a 5-point Likert scale (1 = not at all, 5 = extremely), with higher scores indicating greater threat to the corresponding need. Five items were reverse-coded prior to analysis. This scale indicated good internal consistency in the current study (Cronbach’s α = 0.95).

Manipulation checks were assessed after each Cyberball condition using two items capturing perceived acceptance and rejection. Participants rated the extent to which they felt accepted (“To what extent did you feel accepted by the other players during the game?”) and rejected (“To what extent did you feel rejected by the other players during the game?”) on a 5-point Likert scale (1 = not at all, 5 = extremely). Both items were completed following the inclusion and exclusion conditions, respectively, with higher scores indicating stronger perceived acceptance and rejection.

#### 5.1.3 Data analysis

Data were analyzed in R using linear mixed-effects models fitted with the lmerTest package. Denominator degrees of freedom for fixed effects were estimated using the Satterthwaite approximation, resulting in non-integer *df* values.

Manipulation checks were evaluated using paired-samples t-tests comparing perceived acceptance and rejection across inclusion and exclusion conditions, with effect sizes reported as Cohen’s *d*.

A linear mixed-effects model (LMM) was conducted to examine whether IPD preferences varied as a function of Phase (baseline, inclusion, exclusion) and Target Gender (female vs. male), with random intercepts for participants. *Post hoc* pairwise comparisons were Bonferroni-corrected, and effect sizes are reported as partial eta-squared (η_p_^2^).

A multilevel mediation model was estimated in which experimental condition (0 = inclusion, 1 = exclusion) predicted within-person variation in need threat, which in turn predicted IPD. Random intercepts were specified for participants to account for repeated measurements. Baseline IPD, measured prior to any experimental manipulation, was included as a covariate in supplementary analyses to control for individual differences in initial IPD preference. Indirect effects were evaluated using parametric Monte Carlo simulations (5,000 draws) to obtain confidence intervals.

The multilevel (mixed-effects) moderation models were employed to examine individual differences in the association between need threat and IPD. These models tested whether between-person variables (social identification and rejection sensitivity) moderated the within-person relationship between need threat and IPD via interaction terms. All moderators were grand-mean centered prior to analysis. Significant interactions were probed using simple slopes at ±1 SD. Conditional indirect effects and indices of moderated mediation (IMM) were estimated using Monte Carlo simulations (5,000 draws).

Because social identification and rejection sensitivity may theoretically operate at different stages of the exclusion process, additional moderation analyses were conducted to examine whether these variables moderated the relationship between social exclusion and need threat.

All reported *p*-values are two-tailed, with statistical significance set at *p* < 0.05.

### 5.2 Results

#### 5.2.1 Manipulation checks

Within-participant comparisons of manipulation check items between the inclusion and exclusion conditions were conducted to assess whether the social exclusion manipulation was successful. Paired-samples t-tests showed that, relative to the inclusion condition, participants reported lower perceived acceptance, *t*(395) = 11.91, *p* < 0.001, Cohen’s *d* = 0.60, and higher perceived rejection, *t*(395) = −14.66, *p* < 0.001, Cohen’s *d* = 0.74, in the exclusion condition. These results indicate that participants accurately perceived their exclusion status, confirming the effectiveness of the social exclusion manipulation.

#### 5.2.2 The effects of social exclusion on the regulation of IPD

**Figure 3:**
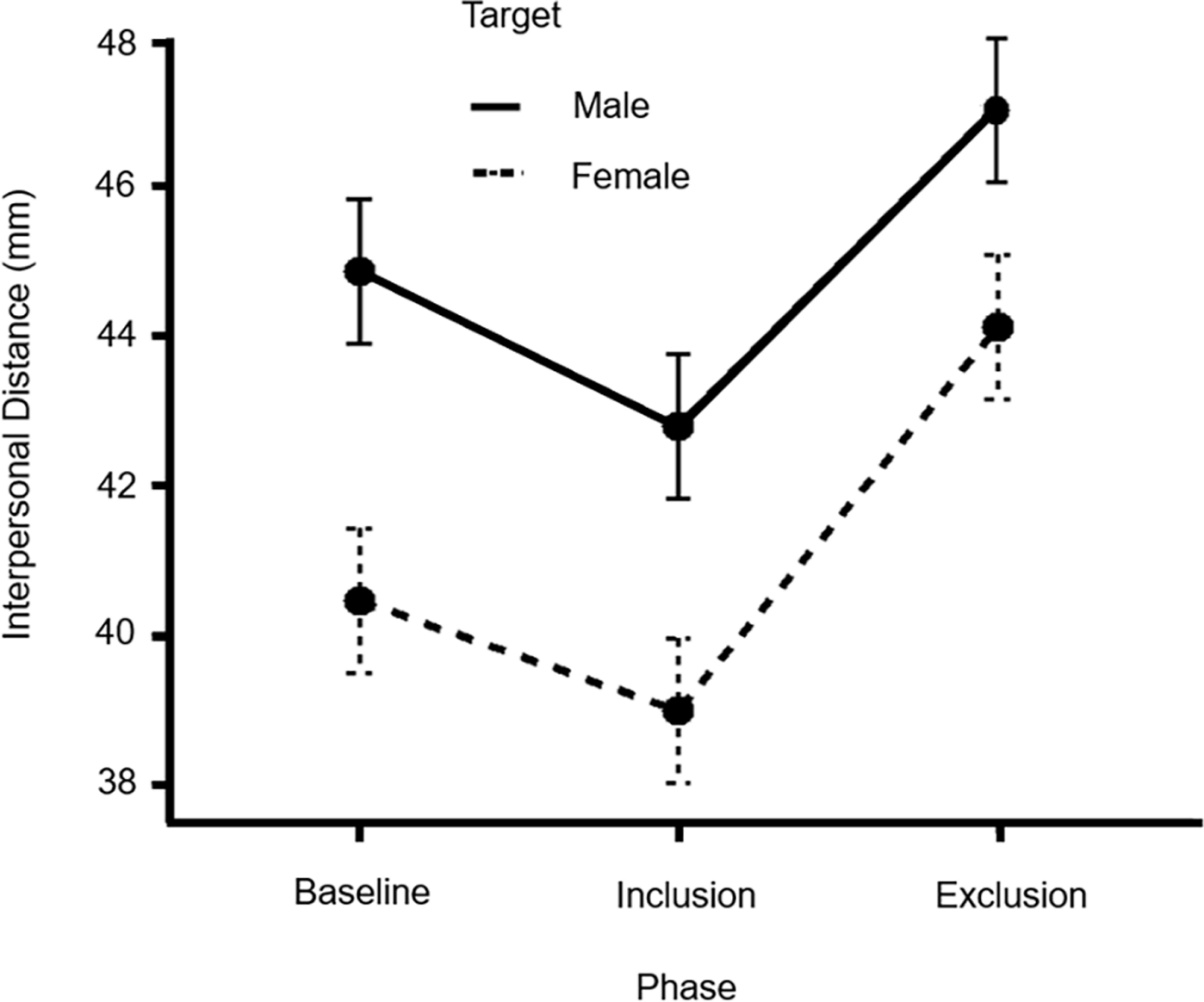
Chosen interpersonal distance as a function of phase and target gender.

A linear mixed-effects model revealed a significant main effect of Phase on IPD, *F*(2, 1980) = 28.94, *p* < 0.001, η_p_^2^ = 0.03. *Post hoc* comparisons indicated that participants maintained greater IPD during the exclusion phase (*M* = 45.58, *SE* = 0.86) than during both the baseline phase (*M* = 42.64, *SE* = 0.86, *p* < 0.001) and the inclusion phase (*M* = 40.87, *SE* = 0.86, *p* < 0.001). Moreover, IPD was significantly reduced during the inclusion phase relative to the baseline phase (*p* = 0.015). A significant main effect of Target Gender also emerged, *F*(1, 1980) = 52.82, *p* < 0.001, η_p_^2^ = 0.03, with greater IPD toward male (*M* = 44.88, *SE* = 0.82) than female targets (*M* = 41.17, *SE* = 0.82). The Phase × Target Gender interaction was not significant, *F*(2, 1980) = 0.67, *p* = 0.514 (see Figure 3).

#### 5.2.3 The psychological mechanisms underlying social exclusion-induced IPD regulation

##### 5.2.3.1 Exclusion-induced need threat as a mediator of IPD regulation

To examine whether need threat mediated the association between social exclusion and IPD, we tested a multilevel mediation model with experimental condition (0 = inclusion, 1 = exclusion) predicting need threat, which in turn predicted IPD. Random intercepts were specified for participants to account for within-person dependence.

Consistent with the manipulation, exclusion significantly increased perceived need threat relative to inclusion (a path), *b* = 0.84, *SE* = 0.06, *t*(396) = 14.23, *p* < 0.001. In turn, higher need threat was associated with greater IPD (b path), controlling for condition, *b* = 6.19, *SE* = 0.46, *t*(518.08) = 13.50, *p* < 0.001. The total effect of social exclusion on IPD was significant (c path), *b* = 4.71, *SE* = 0.68, *t*(396) = 6.98, *p* < 0.001. However, this effect was no longer significant when need threat was included (c’ path), *b* = −0.46, *SE* = 0.69, *t*(430.23) = −0.67, *p* = 0.504.

**Figure 4:**
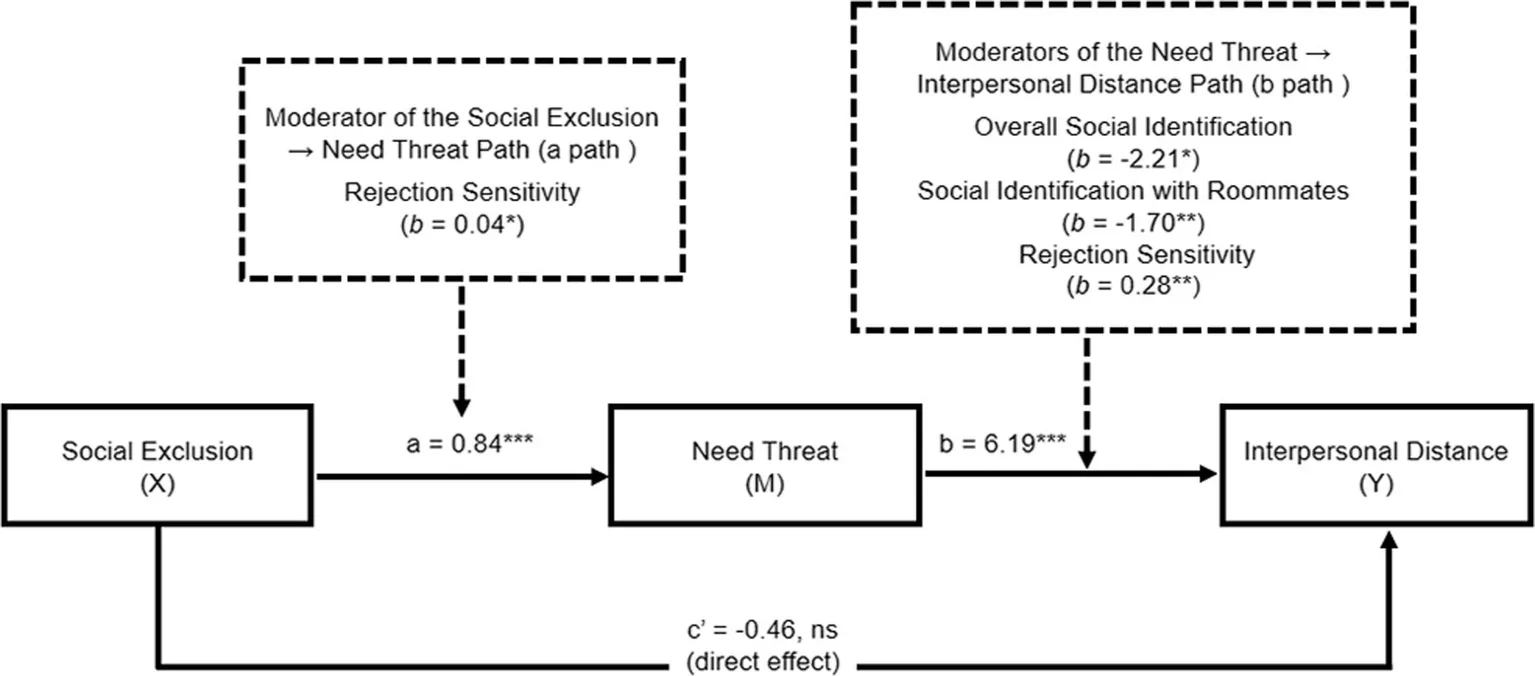
Final moderated mediation model. Unstandardized coefficients are shown for the mediation and moderation pathways. Need threat mediates the association between social exclusion and interpersonal distance. Overall social identification, social identification with roommates, and rejection sensitivity moderate the Need Threat → Interpersonal Distance pathway. Exploratory analyses indicated that rejection sensitivity moderates the Social Exclusion → Need Threat pathway.

Monte Carlo simulations (5,000 draws) further confirmed a robust indirect effect, estimate = 5.18, 95% CI [4.17, 6.24], *p* < 0.001. The direct effect was not significant, 95% CI [−1.82, 0.89], whereas the total effect remained significant, estimate = 4.71, 95% CI [3.36, 6.06], *p* < 0.001. These results are consistent with the mediation of the relationship between social exclusion and IPD through need threat. A graphical representation of the mediation paths can be seen in Figure 4.

##### 5.2.3.2 Fundamental need dimensions as mediators of IPD regulation

To examine whether the observed mediation effect was driven by specific fundamental needs, we conducted exploratory mediation analyses separately for belonging, self-esteem, meaningful existence, and control. Consistent with the primary analysis, significant indirect effects were observed for all four subscales. The magnitude of the indirect effects was broadly comparable across dimensions (belonging: *b* = 4.29, self-esteem: *b* = 4.20, meaningful existence: *b* = 4.13, control: *b* = 3.96; see Supplementary Table S1).

##### 5.2.3.3 Individual differences in the regulation of IPD

To test whether individual differences moderated the association between need threat and IPD preferences, we examined whether social identification (with roommates and family) and rejection sensitivity moderated the within-person association between need threat and IPD.

###### 5.2.3.3.1 Social identification

**Figure 5:**
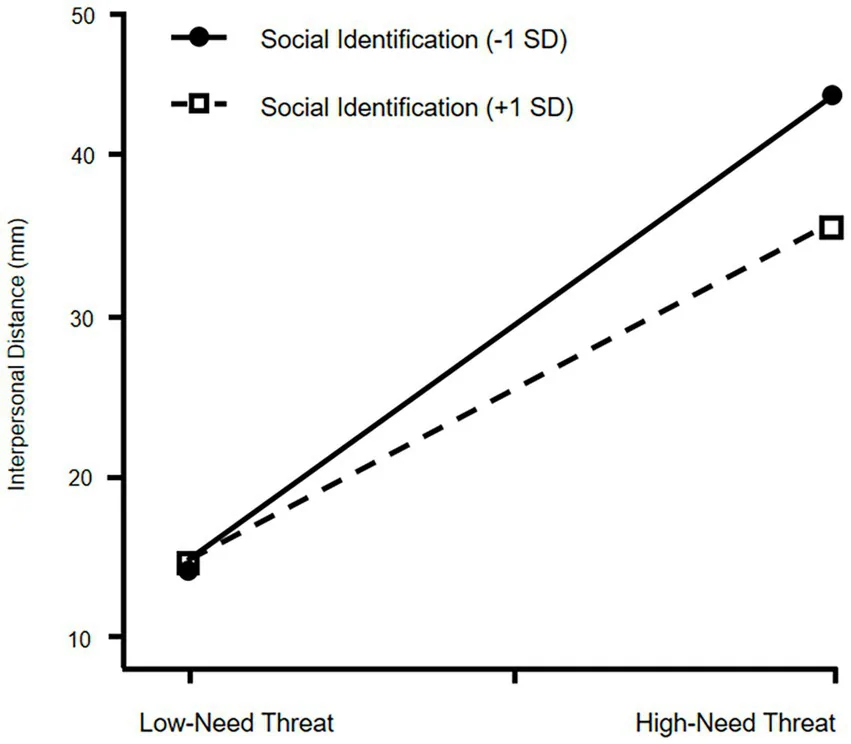
Chosen interpersonal distance as a function of need threat and social identification.

A significant interaction emerged between need threat and SI, *b* = −2.21, *SE* = 1.01, *t*(396) = −2.19, *p* = 0.029. Simple slopes indicated that the association between need threat and IPD was stronger among participants with low SI (−1 SD), *b* = 7.30, *SE* = 0.78, *t* = 9.34, *p* < 0.001, than those with high SI (+1 SD), *b* = 5.21, *SE* = 0.60, *t* = 8.69, *p* < 0.001 (see Figure 5). Monte Carlo estimation of conditional indirect effects further indicated that the mediation effect of need threat was stronger at low SI [95% CI (4.63, 7.71)] relative to high SI [95% CI (3.24, 5.55)]. The index of moderated mediation was significant [IMM = −1.85, 95% CI (−3.58, −0.23)], indicating a negative moderation of the indirect effect by SI.

**Figure 6:**
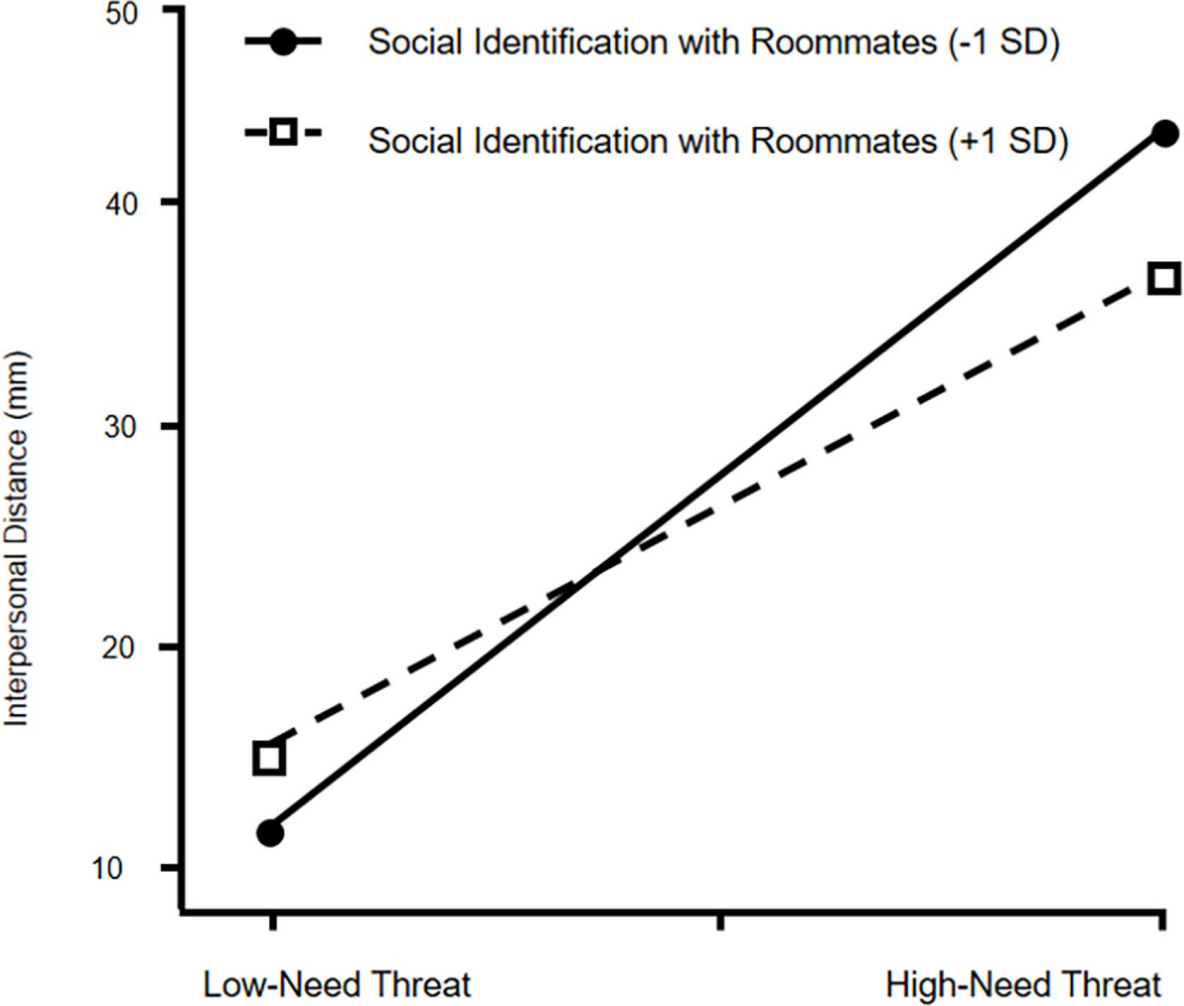
Chosen interpersonal distance as a function of need threat and social identification with roommates.

A similar moderating pattern was observed for SI with roommates, *b* = −1.70, *SE* = 0.57, *t*(396) = −2.98, *p* = 0.003. Simple slopes indicated that the association between need threat and IPD was stronger at low (−1 SD), *b* = 7.36, *SE* = 0.68, *t* = 10.90, *p* < 0.001, than at high (+1 SD) levels of SI with roommates, *b* = 5.15, *SE* = 0.56, *t* = 9.20, *p* < 0.001 (see Figure 6). Conditional indirect effects further indicated that the indirect effect was larger at low levels of SI with roommates [95% CI (4.83, 7.62)] than at high levels of SI with roommates [95% CI (3.26, 5.45)], with a significant index of moderated mediation [IMM = −1.42, 95% CI (−2.40, −0.48)].

In contrast, SI with family did not significantly moderate the association between need threat and IPD (*b* = −1.29, *SE* = 0.85, *t* = −1.51, *p* = 0.131), and the index of moderated mediation was not significant [95% CI (−2.53, 0.31)].

###### 5.2.3.3.2 Rejection sensitivity

**Figure 7:**
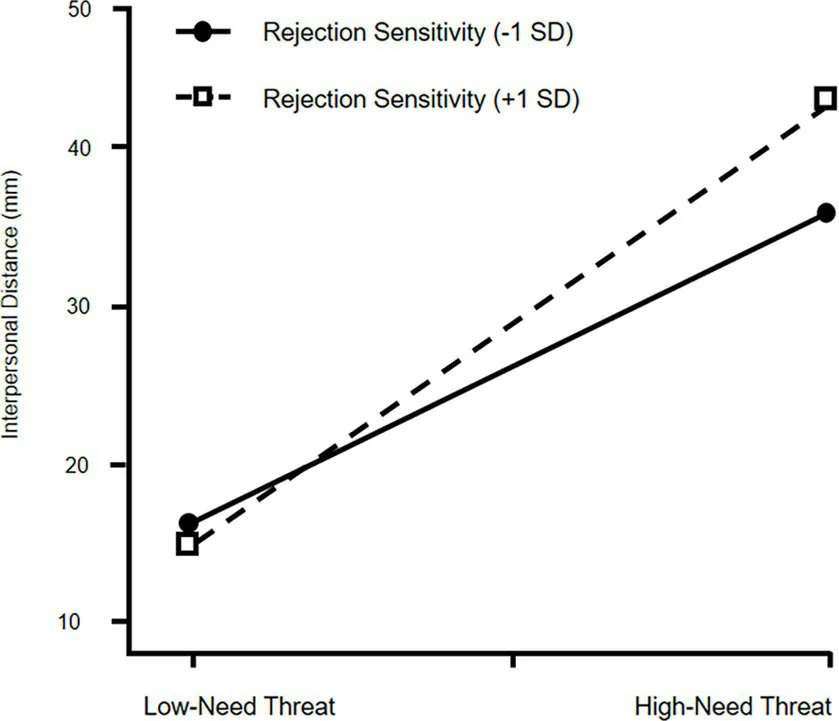
Chosen interpersonal distance as a function of need threat and rejection sensitivity.

RS significantly moderated the association between need threat and IPD, *b* = 0.28, *SE* = 0.09, *t*(396) = 3.00, *p* = 0.003. Simple slopes indicated that the association was stronger at high levels of RS (+1 SD), *b* = 6.85, *SE* = 0.57, *t* = 12.03, *p* < 0.001, compared to low levels of RS (−1 SD), *b* = 4.76, *SE* = 0.63, *t* = 7.52, *p* < 0.001 (see Figure 7). Monte Carlo estimation of conditional indirect effects further indicated that the mediation effect of need threat was stronger at high levels of RS [95% CI (4.54, 6.97)] than at low levels of RS [95% CI (2.82, 5.18)]. The index of moderated mediation was significant [IMM = 0.24, 95% CI (0.08, 0.40)], indicating a positive moderation of the indirect effect by RS.

A graphical representation of the final moderated mediation model is presented in Figure 4.

##### 5.2.3.4 Individual differences in exclusion-induced need threat

To examine whether SI and RS moderated the association between social exclusion and need threat, additional analyses were conducted with need threat as the outcome variable. The interactions involving SI (*b* = 0.24, *p* = 0.057) and SI with roommates (*b* = 0.16, *p* = 0.083) were not statistically significant, suggesting that these variables did not significantly influence the extent to which social exclusion generated need threat. In contrast, RS significantly moderated this association (*b* = 0.04, *SE* = 0.02, *t* = 2.60, *p* = 0.010), indicating that individuals higher in RS experienced a greater increase in need threat following social exclusion. These results are reported in Supplementary Table S2.

##### 5.2.3.5 Supplementary analyses controlling for baseline IPD

To examine whether individual differences in baseline IPD influenced the observed effects, supplementary mediation and moderated mediation analyses were conducted controlling for baseline IPD. The pattern of findings remained highly consistent with the primary analyses. Specifically, the indirect effect of social exclusion on IPD through need threat remained significant [*b* = 4.12, 95% CI (3.27, 5.03), *p* < 0.001], whereas the direct effect remained non-significant [*b* = 0.59, 95% CI (−0.73, 1.90), *p* = 0.380]. In addition, the moderation effects of social identification, social identification with roommates, and rejection sensitivity remained significant after controlling for baseline IPD (see Supplementary Tables S3, S4).

## 6 Discussion

The present study aimed to examine the influence of social exclusion and target gender on interpersonal distance (IPD) preferences within a Chinese sample, using a computerized task (i.e., IVAS; Iachini et al., 2016, 2021; Wei et al., 2024b). In addition to this behavioral approach, we further investigated whether exclusion-induced need threat functions as a psychological process linking social exclusion to IPD preferences. To clarify the extent to which need threat contributes to variation in IPD, we also tested whether individual differences, including social identification (SI) and rejection sensitivity (RS), moderate the association between need threat and IPD preferences.

Findings showed that social exclusion significantly influenced IPD preferences: individuals maintained greater IPD following exclusion, whereas inclusion was associated with a reduction in IPD relative to baseline. On the one hand, the observed expansion of IPD following exclusion contributes to the debate on the relationship between social exclusion and IPD regulation. While some literature indicates that individuals typically exhibited smaller IPD following exclusion (Knowles et al., 2014; Pollatos et al., 2015), others report the opposite trend of larger IPD after exclusion (Chen et al., 2023). Such discrepancies may stem from differences in how social exclusion is operationalized and experienced, underscoring the complexity of context-dependent influences on IPD regulation. On the other hand, this finding refines prior research results suggesting rejection triggers coping responses, such as emotional regulation efforts and behavioral withdrawal during social interactions (Boyes and French, 2009; Chen et al., 2023; Lei et al., 2024; Vangelisti, 2001). These results can be interpreted through the lens of the multimotive model of rejection, which proposes that social exclusion elicits both affiliative and self-protective motives (Smart Richman and Leary, 2009). Although exclusion may provoke social approach behaviors aimed at restoring belonging (Knowles et al., 2014; Pollatos et al., 2015), it also triggers avoidance responses that serve to mitigate potential rejection-related threat and emotional distress. The increased IPD following exclusion thus reflects the predominance of self-protective regulation, whereby individuals strategically increase interpersonal space to manage perceived social threat and restore psychological safety.

This pattern is further supported by proxemics theories (Hall and Hall, 1966), which posit that environments perceived as socially threatening or uncertain are typically associated with enlarged IPD, whereas contexts characterized by safety and comfort promote closer interpersonal proximity (Candini et al., 2019, 2021; Dosey and Meisels, 1969; Lloyd, 2009). From this perspective, social exclusion is typically perceived as a salient cue of social threat, thereby eliciting distance-increasing regulatory responses. Conversely, the reduction in IPD following inclusion aligns with belongingness regulation frameworks, which propose that positive social engagement restores psychological belonging, interactional comfort, and interpersonal security (Baumeister and Leary, 1995; Williams, 2009), thereby increasing tolerance for reduced interpersonal space and more open to social approach (Hall and Hall, 1966; Candini et al., 2019, 2021; Dosey and Meisels, 1969; Lloyd, 2009). These findings suggest that IPD operates as a context-sensitive behavioral regulatory mechanism that flexibly reflects shifts between avoidance-oriented and affiliation-oriented responses across exclusionary and inclusive social experiences.

In addition, participants maintained greater IPD from male than from female targets. This finding is broadly consistent with previous research showing that IPD tends to be greater toward male than female targets (Beck and Ollendick, 1976; Iachini et al., 2016; Wei et al., 2024b). However, because target gender was not a primary theoretical focus of the present study and no specific hypotheses regarding gender differences were proposed, this finding is best regarded as exploratory. Moreover, the absence of a significant interaction between phase and target gender suggests that the effect of social exclusion on IPD did not differ as a function of target gender. Future research could further investigate the mechanisms underlying gender-related differences in IPD and examine how these processes may operate in the context of social exclusion.

The present results revealed that the effect of social exclusion on IPD preferences operated primarily through increased threat to fundamental psychological needs. Specifically, exclusion elevated perceived need threat, which, in turn, was associated with a preference for greater IPD. This pattern is consistent with the temporal need-threat model, which posits that social exclusion undermines fundamental psychological needs by removing cues of social acknowledgment, a critical indicator of interpersonal inclusion (Boyes and French, 2009; Williams, 1997, 2009; Williams et al., 2000; Zadro et al., 2004). The absence of such acknowledgment may signal social devaluation, thereby undermining individuals’ sense of belonging (Baumeister and Leary, 1995). This perceived devaluation further conveys implicit negative evaluation that may be internalized as reduced self-worth, leading to diminished self-esteem (Chen et al., 2023). In parallel, exclusion limits individuals’ perceived control over social interactions, as the inability to elicit responses from others reduces their sense of interpersonal agency. This loss of interpersonal agency, coupled with the experience of being ignored, fosters a sense of social invisibility, thereby threatening individuals’ sense of meaningful existence (Williams, 2007, 2009).

Results further demonstrated that the need threat was behaviorally expressed through the regulation of IPD. Specifically, heightened need threat was associated with reduced tolerance for close interpersonal proximity. These patterns accord with proxemics theory and extend prior evidence showing that individuals tend to maintain greater IPD under conditions of perceived threat (Candini et al., 2019; Dosey and Meisels, 1969; Hall and Hall, 1966; Lloyd, 2009; Wei et al., 2024b). This tendency is likely linked to shifts in the appraisal of threatened fundamental needs, which heighten individuals’ sensitivity to the interactional demands and uncertainties inherent in social encounters. This heightened sensitivity may, in turn, increase the perceived risk and psychological cost of close interpersonal engagement, thereby reducing individuals’ willingness to tolerate close-range contact. Within this regulatory context, individuals may adjust interpersonal boundaries by increasing distance from others to limit interpersonal exposure. These findings suggest that need threat is not merely an internal psychological state but also a determinant of observable social behavior, shaping how individuals regulate interpersonal proximity. In this sense, IPD operates as a context-sensitive indicator of boundary regulation in response to threatened fundamental needs.

This distancing behavior can also be interpreted within an embodied cognition framework, which posits that cognitive processes are grounded in the dynamic integration of sensory input, affective responses, and motor actions, and are continuously shaped by bodily interactions with the environment (D’Angelo et al., 2017; De Vignemont, 2011; Paladino et al., 2010; Schnall, 2011). From this perspective, threats to fundamental psychological needs may be experienced as embodied affective states (e.g., heightened threat sensitivity and physiological arousal), which in turn promote defensive action tendencies, resulting in a preference for greater physical distance. Increased IPD may therefore function as a behavioral strategy through which individuals restore a sense of safety and affective equilibrium, reflecting the coordinated influence of emotional and motor processes on interpersonal space regulation. This perspective complements cognitive-evaluative accounts of IPD regulation by emphasizing the embodied nature of defensive social behavior.

Although the present findings support the role of need threat in explaining exclusion-induced increases in IPD, alternative psychological processes may also contribute to distancing behavior. Prior research has frequently associated greater IPD with heightened anxiety, perceived threat, negative affect, and social unease (Lloyd, 2009; Perry et al., 2013; Wei et al., 2024a). These responses may reflect more general reactions to negative emotional states rather than processes unique to social exclusion. From this perspective, the observed preference for greater IPD may arise from a broader constellation of affective and threat-related responses that operate alongside need threat. Rather than representing competing explanations, these processes may jointly contribute to interpersonal distancing following exclusion. Future research could further clarify their relative contributions by examining multiple psychological mechanisms simultaneously.

Beyond these broader psychological processes, it is also important to consider whether the observed mediation effect reflects a generalized need-threat response or is primarily driven by specific fundamental needs. Exploratory analyses at the subscale level indicated that all four fundamental needs significantly mediated the association between social exclusion and increased IPD. Although the magnitude of the indirect effects varied slightly across subscales, with belonging showing the strongest effect followed by self-esteem, meaningful existence, and control, the effects were broadly comparable in magnitude. These findings suggest that social exclusion simultaneously threatened multiple fundamental needs, each of which was associated with greater IPD. Thus, the observed mediation appears to reflect a broad need-threat response rather than the influence of a single threatened need. At the same time, because the four needs are conceptually distinct, aggregating them into a composite score may obscure subtle differences in their relative contributions. Future research may therefore benefit from examining individual need dimensions separately to determine whether specific needs play a more prominent role under particular social conditions.

These exploratory findings may also provide insight into why social exclusion was associated with increased interpersonal distance rather than the interpersonal approach often emphasized by the Social Monitoring System (SMS). Although threats to belonging are typically considered central to exclusion-induced reconnection efforts (Gardner et al., 2000; Maner et al., 2007), belonging did not emerge as a uniquely stronger pathway linking exclusion to IPD. Instead, threats to self-esteem, control, and meaningful existence exhibited mediation effects of a broadly similar magnitude. This pattern suggests that the psychological consequences of exclusion extended beyond belonging-related concerns and involved multiple threatened needs simultaneously. Consequently, exclusion may evoke not only affiliative motivations aimed at restoring social connections, but also concerns about potential social risks, diminished interpersonal control, and further negative social outcomes. These processes are not necessarily mutually exclusive and may operate concurrently. In the present study, the resulting behavioral response was associated with greater IPD, suggesting that when exclusion threatens multiple fundamental needs simultaneously, interpersonal caution and boundary regulation may become more salient than immediate social approach.

The present findings showed that the association between need threat and IPD preferences was moderated by SI. Specifically, this association was attenuated among individuals with high SI. This result refines previous studies demonstrating the regulatory role of SI in shaping social behavior and buffering the impact of adverse social experiences (Lee et al., 2015; Wei et al., 2024a; Van Dick et al., 2005). This moderating effect can be understood in terms of the extent to which exclusion-induced need threat is incorporated into self-relevant processing, a process fundamentally shaped by how individuals define the self (Tajfel and Turner, 2004; Turner et al., 1987). Individuals with high SI tend to derive a stable sense of belonging and self-definition from enduring group memberships, thereby reducing reliance on immediate interpersonal feedback. Although exclusionary cues may signal situational non-acceptance, such individuals are less likely to construe these experiences as indicative of enduring relational devaluation (Barlow et al., 2010; Li and Jiang, 2018). Consequently, exclusion-induced need threat is less incorporated into self-evaluative processing, reducing its self-relevance and decreasing the likelihood of engaging in self-protective behaviors such as increasing IPD. In contrast, individuals with low SI rely more heavily on situational interpersonal cues for self-definition, rendering exclusion-induced need threat more likely to be internalized. This heightened self-relevance increases the perceived personal significance of the threat and strengthens its appraisal as self-threatening, thereby facilitating the defensive boundary regulation, reflected in increased IPD to protect the self from further social harm.

A parallel moderating pattern emerged for identification with roommates, but not for identification with family. Specifically, identification with roommates attenuated the association between exclusion-induced need threat and IPD, whereas identification with family did not exhibit a comparable buffering effect. This pattern extends previous research suggesting that peer-based belonging plays a central role in shaping interpersonal regulatory behaviors (Maunder, 2018; Savolainen et al., 2019; Seo et al., 2025), and it is consistent with social identity theory, which posits that the regulatory impact of group identification depends on the situational salience of the group at the moment of threat (Tajfel and Turner, 2004, 2010; Turner et al., 1987).

This divergent pattern can be attributed to differences in the contextual accessibility of these group memberships. For university students, roommate relationships constitute an interactionally proximal and structurally embedded social context, characterized by frequent contact and ongoing interdependence (Erb et al., 2014). As such, roommate-based identification is readily activated within the immediate interpersonal context in which exclusion occurs. When the need threat is elevated, this accessible source of belonging can be recruited to provide psychological security, thereby reducing the need to expand IPD. In contrast, although identification with family is often central to the self-concept and emotionally significant, it is less directly implicated in the immediate interactional context. In university settings, family relationships are typically geographically distal and less embedded in ongoing social exchanges. Consequently, family-based identification is less likely to be situationally activated at the moment of threat and is therefore less effective in shaping real-time interpersonal regulation, resulting in a comparatively limited buffering effect on the association between need threat and IPD. These findings suggest that the buffering effect of SI on need threat-induced distancing depends primarily on the situational relevance of the identified group.

Complementing the buffering effects observed for SI, the present results further revealed that RS strengthened the association between fundamental need threat and IPD preferences. Specifically, this association was more pronounced among individuals high (vs. low) in RS. This pattern aligns with the RS framework and advances prior research by showing that individuals high in RS tend to exhibit social avoidance, such as reduced physical contact and diminished social engagement (Meehan et al., 2018; Schaan et al., 2020; Zimmer-Gembeck, 2015). This moderating effect can be explained by heightened sensitivity to interpersonal threat cues and negatively biased threat appraisal (Downey and Feldman, 1996; London et al., 2007). When exclusion induces momentary need threat, individuals high in RS are more likely to interpret such experiences and subsequent social interactions as indicative of a persistent and generalized risk of rejection, rather than as a transient, context-specific disruption. This biased appraisal process heightens vigilance toward potential threat cues and amplifies anticipatory concerns about further exclusion (Romero-Canyas et al., 2010; Williams, 2009). In turn, these processes are likely to activate defensive motivational responses aimed at minimizing further rejection, which are behaviorally reflected in increased IPD. In this sense, RS operates as a dispositional threat amplifier that strengthens the behavioral expression of exclusion-induced need threat in interpersonal distancing.

The present findings further provide insight into how individual difference factors operate across the exclusion process. Although both variables moderated the association between need threat and IPD, significant moderation of the relationship between social exclusion and need threat emerged only for RS. Specifically, individuals higher in RS experienced a stronger increase in threatened needs following exclusion than those lower in RS, whereas the corresponding effects for SI did not reach conventional levels of statistical significance, although both effects were in the predicted direction. These findings suggest that SI primarily functions as a buffering resource at the behavioral response stage, influencing how individuals respond to threatened needs once they have been activated. In contrast, RS appears to exert a broader influence across multiple stages of the exclusion process. Specifically, it may operate earlier by amplifying the perception of social threat and may continue to shape subsequent behavioral responses. More broadly, these findings highlight the importance of distinguishing between factors that influence the perception of social threat and those that shape responses to it.

These findings may also help explain previously inconsistent findings regarding the relationship between social exclusion and IPD. Prior research has reported both interpersonal approach and interpersonal avoidance following exclusion, suggesting that exclusion does not produce a uniform pattern of social behavior. The present results indicate that the behavioral consequences of exclusion-induced need threat are shaped by individual differences in the appraisal and regulation of social threat. Specifically, stronger SI appeared to buffer the translation of need threat into defensive interpersonal responses, whereas higher RS strengthened this tendency. From this perspective, SI and RS may serve as important boundary conditions that help explain why similar exclusion experiences lead to different patterns of IPD regulation across individuals and studies, thereby contributing to the integration of previously inconsistent findings in the literature.

Additional analyses further demonstrated the robustness of the present findings. Specifically, the mediation and moderated mediation effects remained significant after controlling for baseline IPD, suggesting that the observed associations reflect responses to social exclusion rather than stable individual differences in IPD preference.

### 6.1 Limitations and future directions

Despite advancing understanding of the mechanisms underlying IPD regulation in the context of social exclusion, this study has several limitations that should be addressed in future research. First, this study was conducted within a specific cultural context using a sample of Chinese college students. Although this design enabled controlled examination of the proposed mechanisms, the findings may not be readily generalizable to individuals from different cultural or age groups. Future research should therefore examine whether the observed effects of social exclusion on IPD, along with the mediating role of need threat and the moderating roles of SI and RS, can be replicated across more diverse cultural and developmental backgrounds. In addition, the sample was predominantly male, which may have influenced the observed target-gender effect and limits the generalizability of gender-related findings. Because relatively few female participants were included, it remains unclear whether a similar pattern would emerge in more gender-balanced samples. Future studies are needed to determine the robustness of this finding across populations with different gender compositions. Second, although the Cyberball paradigm provides a well-established and controlled method for manipulating social exclusion, it represents a relatively minimal and transient form of social threat. As such, it may not fully capture the complexity and intensity of exclusion experiences encountered in real-world social interactions. Future studies could extend these findings by employing more ecologically valid paradigms or longitudinal designs to investigate how chronic or naturally occurring exclusion shapes IPD regulation over time. Third, the Cyberball conditions were administered in a fixed sequence, with inclusion always preceding exclusion. Although this design was intended to minimize potential carry-over effects of exclusion, order effects cannot be completely ruled out. Nevertheless, the observed changes in need threat and interpersonal distance were consistent with the expected effects of social exclusion. Future studies employing counterbalanced designs would help further establish the robustness of the present findings. Finally, while the current study provides valuable insight into IPD preference toward strangers, it does not address potential differences in IPD toward familiar individuals, such as friends or family members, whose social dynamics may differ. Future research could further explore how social exclusion influences IPD through need threat, as well as the moderating roles of SI and RS, not only in interactions with strangers but also within close interpersonal relationships. Such work would broaden understanding of IPD regulation across a wider range of social contexts and relational settings, thereby further enhancing the generalizability of the findings.

## 7 Conclusion

This study found that social exclusion increased individuals’ preferences for greater interpersonal distance through heightened fundamental need threat. Moreover, the association between need threat and interpersonal distance was stronger among individuals lower in social identification and higher in rejection sensitivity. These findings indicate that interpersonal distance reflects not only situational responses to social threat, but also broader processes of interpersonal safety management and social adaptation. Overall, the present study advances understanding of the psychological mechanisms through which social exclusion influences interpersonal behavior and highlights how individual differences shape interpersonal responses to exclusion-induced need threat, with potential implications for interventions aimed at reducing maladaptive interpersonal responses following exclusion.

## Data Availability

The raw data supporting the conclusions of this article will be made available by the authors, without undue reservation.
