# Supplementary material for: Social exclusion increases interpersonal distance through need threat: buffering and amplifying roles of social identification and rejection sensitivity
Source: Front Psychol. 2026 Sep 8;17:1881465. doi: 10.3389/fpsyg.2026.1881465 (PMC13595841; doi:10.3389/fpsyg.2026.1881465)
Supplement: Supplementary file 1 [file Supplementary_File_1.docx]

**Supplementary Materials**

**Table S1**

**Exploratory mediation analyses of the four need threat subscales in the association between social exclusion and interpersonal distance**

| **Mediator** | **X → M (β)** | ***p*** | **M → Y (β)** | ***p*** | **Direct (c’ β)** | ***p*** | **Indirect (β)** | **95% CI** | ***p*** |
| --- | --- | --- | --- | --- | --- | --- | --- | --- | --- |
| Belonging | 0.98 | <.001 | 4.39 | <.001 | 0.42 | .549 | 4.29 | [3.35, 5.30] | <.001 |
| Self-esteem | 0.73 | <.001 | 5.77 | <.001 | 0.51 | .472 | 4.20 | [3.29, 5.18] | <.001 |
| Meaningful existence | 0.83 | <.001 | 4.97 | <.001 | 0.59 | .383 | 4.13 | [3.22, 5.09] | <.001 |
| Control | 0.81 | <.001 | 4.90 | <.001 | 0.75 | .276 | 3.96 | [3.08, 4.91] | <.001 |

**Note.** X = Social Exclusion condition (Inclusion vs. Exclusion); Y = Interpersonal Distance (IPD). Mediators are the four subscales of the Need Threat Scale. Indirect effects were estimated using Monte Carlo simulations (5,000 draws) and were all significant at p < .001.

**Table S2**

**Moderation analyses of social identification and rejection sensitivity on the association between social exclusion and need threat**

| **Moderator** | **Interaction (condition × moderator)** | ***β*** | ***SE*** | ***t*** | ***p*** |
| --- | --- | --- | --- | --- | --- |
| SI | condition × SI | 0.24 | 0.12 | 1.91 | .057 |
| SI with Roommates | condition × SI with Roommates | 0.16 | 0.09 | 1.74 | .083 |
| RS | condition × RS | 0.04 | 0.02 | 2.60 | .010 |

**Note.** SI = Social Identification; RS = Rejection Sensitivity. Models examined whether individual differences moderated the association between experimental condition (0 = inclusion, 1 = exclusion) and need threat. Positive interaction coefficients indicate that the increase in need threat from inclusion to exclusion was greater at higher levels of the moderator.

**Table S3**

**Supplementary mediation analysis controlling for baseline interpersonal distance**

| **Effect** | **Estimate** | **95% CI** | ***p*** |
| --- | --- | --- | --- |
| a path (Social Exclusion → Need Threat) | 0.84 | — | < .001 |
| b path (Need Threat → IPD) | 4.93 | — | < .001 |
| Direct effect (c′) | 0.59 | [-0.73, 1.90] | .38 |
| Indirect effect (a × b) | 4.12 | [3.27, 5.03] | < .001 |
| Total effect (c) | 4.71 | [3.40, 5.99] | < .001 |

**Note.** Baseline interpersonal distance was included as a covariate in all mediation models. Indirect effects were estimated using Monte Carlo simulations (5,000 draws).

### Table S4

### Supplementary moderation analyses controlling for baseline interpersonal distance

| **Moderator** | **Interaction *β*** | ***SE*** | ***t*** | ***p*** |
| --- | --- | --- | --- | --- |
| Overall Social Identification | -2.21 | 1.01 | -2.19 | .029 |
| Social Identification with Roommates | -1.70 | 0.57 | -2.98 | .003 |
| Rejection Sensitivity | 0.28 | 0.09 | 3.00 | .003 |

**Note.** Baseline interpersonal distance was included as a covariate in all moderation models.
